# The Flow Rate of the Condenser Cooling Water in the Distillation Process Increases the Quality and Quantity of Patchouli Oil

**DOI:** 10.1155/2024/9844242

**Published:** 2024-02-15

**Authors:** Husein Smith, Muhammad Assagaf, Rosniyati Suwarda, Agus Budiyanto, Himawan Adinegoro, Lamhot Parulian Manalu, Rienoviar, Rosniati, Feri Manoi, Justus Elisa Loppies, Sitti Ramlah, Sunarmani, Siti Mariana Widayanti, Setyadjit, Tri Marwati, Nurdi Setyawan, Rudy Tjahjohutomo, Enrico Syaefullah, Risfaheri, Abdullah Bin Arif

**Affiliations:** ^1^Research Center for Agroindustry, National Research and Innovation Agency, Central Jakarta, Indonesia; ^2^Research Center for Food Technology and Processing, National Research and Innovation Agency, Central Jakarta, Indonesia; ^3^Research Center for Appropriate Technology, National Research and Innovation Agency, Central Jakarta, Indonesia; ^4^Indonesian Agricultural Engineering Polytechnic, Ministry of Agriculture, South Jakarta, Indonesia

## Abstract

Indonesia is an important essential oil-exporting country globally, where 40 types of essential oils have been traded on the international market and are products of Indonesia. However, the quality and quantity of patchouli oil produced in Indonesia are still low. Most essential oil processing units use simple or traditional technology and generally have limited production capacity. This study aimed to obtain the optimum water flow rate in a condenser system for patchouli oil production in Maluku, Indonesia. Patchouli oil extraction from fresh patchouli leaves and twigs was carried out by increasing the condenser water discharge rate. Patchouli oil extraction with a condenser cooling water discharge treatment of 1.74 L/min and drying time for 5 days produced the highest patchouli oil yield of 1.4%. The greater the condenser water discharge rate, the better the yield and accumulation of patchouli oil recovery obtained. In addition, based on the results of the analysis of the composition of patchouli oil compounds with GCMS, it can be seen that 13 compounds can be detected in patchouli oil. The three main components of patchouli oil in all condenser cooling water treatments were alpha-guaiene, delta-guaiene, and patchouli alcohol. Considering the results of all parameters mentioned above, the treatment of the condenser cooling water discharge of 1.74 L/min and drying time for 5 days increases the quality and quantity of patchouli oil.

## 1. Introduction

Indonesia is an agricultural country with extraordinary natural wealth and abundance of various types of plants. As one of the most critical essential oil-exporting countries in the world, 40 types of essential oils traded on the international market are products of Indonesia [1]. Essential oil-producing plants that have market opportunities include patchouli. The *Pogostemon* genus is estimated to have 40 species, but patchouli is the only species with market opportunities [2]. Patchouli (*Pogostemon cablin*) comes from the Lamiaceae family. It is one of the essential oil-producing plants from tropical Asia. It is cultivated in the Caribbean, China, India, Indonesia, and the Philippines, as well as in several states of Karnataka, Gujarat, and Assam in India [3, 4]. Patchouli oil obtained by distilling the leaves of *Pogostemon cablin* Benth contains more than 24 different sesquiterpenes [5]. This oil is widely used in the perfume, soap, pharmaceutical, and cosmetic industries [6] or as aromatherapy which can heal physically, mentally, and emotionally [7]. The addition of oil with the patchouli alcohol component to perfume can maintain the aroma of perfume for a long time because of its function as a binder that cannot be replaced by any oil [8, 9], so it is essential in making perfume [10]. Patchouli oil also has pharmacological effects, including antibacterial, antifungal, antiemetic [11, 12], anti-influenza [13], antioxidant [14], antimicrobial [15, 16], irritation, skin inflammation, bioinsecticide [17], and anticancer activities [18–20]. However, the quality and quantity of patchouli oil produced in Indonesia still need improvement. Most essential oil processing units use simple or traditional technology and generally have limited production capacity.

One factor affecting patchouli oil production is postharvest handling and extraction methods. In Indonesia, oil is extracted from patchouli leaves and twigs by distillation [21]. Different distillation methods affect the oil yield [22]. Steam distillation is a traditional method for obtaining patchouli oil [23, 24]. The hydrodistillation process takes a long time and will affect the degradation of patchouli oil molecules [25]. One technology that is proven to increase oil yield through distillation is the cooling condenser technology. The cooler (water condenser) is a vital heat exchanger in refining patchouli oil because it condenses the steam [26]. The low oil yield can be caused by various factors, including the performance of the heat exchanger (condenser) that could be more optimal. The effectiveness of the condenser is influenced by several factors, including condenser design, condenser water temperature, material conductivity, and other factors [27]. Color change, chemical-physical properties, essential oil yield, and distillation speed are affected by the water temperature in the condenser because it is related to the speed of steam condensation in the cooling pipe. Several related studies have shown that water as a condenser coolant cools the condenser faster than air [28]. The performance of the shell and tube condenser is influenced by the heat exchanger's design, the fluid's mass flow rate, the conductivity value of the heat exchanger material, the insulation density, and the temperature and ambient temperature [29]. The research results of Ahmad and Anis [30] show that the greater the cooling water discharge in the condenser, the higher the yield of distilled pine resin oil and the greater the heat transfer rate, which causes the condenser to cool [31]. On the other hand, if the cooling water velocity decreases, the condenser water temperature increases, which affects the heat transfer rate [32].

Patchouli oil refining in Maluku, Indonesia, uses a water steam distillation system and a traditional heat exchanger (condenser). This refining resulted in a low yield of patchouli oil due to the not-yet-optimal distillation process technology and the performance of the heat exchanger (condenser) device. For this reason, obtaining a better distillation system is necessary to increase the yield and quality of patchouli oil. Therefore, this study aims to obtain the optimum water flow rate in a condenser system for patchouli oil production in Maluku, Indonesia.

## 2. Materials and Methods

### 2.1. Plant Materials

Fresh patchouli leaves and twigs used in this study were Aceh patchouli (*Pogostemon cablin* Benth) taken from patchouli oil farmers/refiners from Taeno village, Keranjang village, Ambon District, Maluku Province, Indonesia. Leaves and twigs of *Pogostemon cablin* were collected in June 2021 and stored at room temperature.

### 2.2. *Pogostemon cablin* Extraction

**Figure 1:**
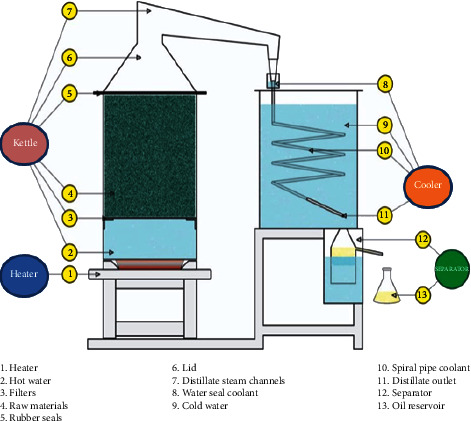
Patchouli oil distillation equipment.

Patchouli leaves and twigs that were cut into smaller pieces were dried in the sun for two days and dried under the shade for 5 (*B*_1_), 6 (*B*_2_), and 7 days (*B*_3_) and then distilled using the steam and water distillation method. The treatment of condenser cooling water discharge was based on the study by Smith et al. [33] with modifications, namely, 1.74 (*A*_1_), 1.08 (*A*_2_), and 0.48 liter/minute (*A*_3_). The distilled apparatus was made of stainless steel with a capacity of 8 kg. The distillation duration was based on the amount of patchouli raw material being distilled, and distillation was stopped if no oil came out simultaneously with the condensate water (±3 hours) [34]. The response variables measured were the oil yield, condensate flow rate, condenser water temperature, and patchouli alcohol content. The term condensate flow rate in this study is the rate of steam fluid condensing in the condenser pipe that comes out in the form of a mixture of patchouli oil and water. In addition, the content of compounds in patchouli oil was measured using GCMS. The equipment used includes steam-water distillation equipment consisting of a boiler and condenser, stove (as heater), separator, and laboratory equipment for patchouli oil testing. In the condenser, cooling water was circulated using a circulation pump. In addition, the equipment used in this study included a distillation kettle with a filling volume of 0.023 m^3^ of patchouli leaves, a water-cooled condenser with a spiral-shaped heat exchanger type with a cold fluid volume of 0.022 m^3^, and a separator (Figure 1). The research was conducted at the Ambon Industrial Standardization and Research Institute, Indonesia.

### 2.3. Determination of Patchouli Alcohol in Patchouli Oils

Patchouli alcohol was determined according to the method described by Kongkathip et al. [15]. The sample volume was 2 *μ*L. Patchouli alcohol concentration was calculated on the basis of the linear calibration function. The content of patchouli alcohol was expressed as grams per 100 g of dry weight.

### 2.4. Patchouli Oil Component Identification

A gas chromatography-mass spectroscopy (GCMS) technique was used to analyze the chemical composition of patchouli oil. GCMS analysis used gas chromatograph Merck Agilent Technologies Type 7890 B with a nonpolar column, namely, HP-1 MS (methyl siloxane), with specifications of 30 m × 25 *μ*m × 0.25 *μ*m. The oven temperature was held constant at 60°C for 1 min and then increased to 220°C with a temperature increase rate of 30°C/min. The final oven temperature was then kept constant for 4 min. The injector, transfer line, and detector temperatures were 250, 240, and 230°C, respectively. Ionization energy was 70 eV, and the flow rate of carrier gas (helium) was 1 ml/min. The samples (1% v/v in hexane) were injected into GC by split mode with a split ratio of 1/100. Patchoulol identification and other volatile compounds were based on comparing the mass spectra obtained in the gas chromatograph with those obtained from the GCMS library and literature [35–38].

### 2.5. Statistical Analysis

Experiments were performed using a completely randomized factorial design with three replications. Means were compared by one-way analysis of variance and Duncan's multiple range test (DMRT) at a 5% significance level. Data are presented as the mean.

## 3. Result and Discussion

### 3.1. The Condensate Flow Rate and Condenser Water Temperature

The treatment of condenser cooling water discharge and the drying time of patchouli affected the condensate flow rate. Distillation of patchouli leaf oil, which was dried under the shade for five days (B1), resulted in a condensate flow rate of 34.67, 29.33, and 24.33 ml/min for the treatment of A1, A2, and A3 condenser cooling water discharge, respectively (Table 1). The treatment of the condenser cooling water discharge of 1.74 L/min and drying for five days on patchouli leaves resulted in the highest condensate flow rate, which was 2.17-fold faster than the condenser cooling water discharge of 0.48 L/min and drying for seven days (general conditions to produce patchouli oil in farmers/refiners). The decrease in the condensate flow rate is due to the reduced condenser cooling water discharge caused by the difference in the velocity of steam fluid condensing into the liquid fluid in the condenser pipe. The high discharge of condenser cooling water causes a faster heat transfer of the steam fluid in the condenser pipe. The higher the condenser cooling water discharge, the faster the process of condensing the liquid vapor from the condenser into a liquid (a mixture of oil and water). Cooling water discharge affects the condenser's heat transfer rate [27]. The faster the flow rate of the coolant, the faster the heat transfer rate occurs in the condenser [29].

Determination of condenser water temperature showed that there were differences in each treatment of condenser water discharge and drying time of patchouli leaves. The lowest condenser water temperature was 30.56°C in the patchouli oil distillation treatment. The condenser cooling water discharge was 1.74 L/min with the leaves drying time for 5 days (Table 2). The smaller the cooling water discharge, the higher the condenser water temperature. This is caused by differences in the heat transfer of water contained in the condenser shell in each treatment of cooling water discharge. The condenser water temperature is affected by the discharge of the condenser cooling water in the essential oil production process [29, 30]. The effect of temperature on the yield is detrimental, where under isobaric conditions, an increase in temperature reduces the density of supercritical CO_2_, thereby reducing the solubility of the fluid [39]. However, a higher temperature increases the vapor pressure of the solute, making oil more accessible to extract [40]. In addition, an increase in temperature causes antioxidants and bioactive compounds that are sensitive to an increase in temperature to be degraded [41]. This harms the antioxidant activity. Therefore, in this study, the condenser cooling water discharge treatment of 1.74 and 1.08 L/min inhibited the increase in condenser water temperature.

### 3.2. Patchouli Oil

The results of the distillation of patchouli oil using steam and water distillation and with a shell and tube-type heat exchanger (condenser) showed differences in the yield of patchouli oil produced in the condenser release treatment and the drying time of patchouli leaves. The greater the condenser water discharge during distillation, the greater the patchouli oil yield (Table 3). However, the longer the leaves were exposed to the sun, the lower the yield of patchouli oil (Table 3). Distillation of oil from patchouli leaves in the treatment of condenser cooling water discharge of 1.74 l/min and drying time for 5 days resulted in a yield of patchouli oil of 1.4%, while the distillation of dried leaf oil for 5 days with condenser water discharge produced a higher yield of patchouli oil was 0.37% and 1.06% for the treatment of condenser water discharge of 1.08 and 0.48 l/min, respectively (Table 3). The difference in the yield of patchouli oil in each treatment showed the contribution of the condenser cooling water debit and drying of the leaves to the patchouli oil yield. The condenser water discharge significantly affects the amount of pine resin oil produced; the higher the condenser cooling water discharge, the higher the pine resin oil produced [27]. The higher the condenser cooling water discharge, the faster the heat transfer rate from the hot steam fluid in the tube/pipe to the cooling water in the shell. Water-cooled condensers cool the condensers faster than air-cooled condensers [28].

Patchouli alcohol is the main component of patchouli oil [5, 42]. The alcohol content of patchouli oil is usually used as a reference for assessing the quality of patchouli oil [43]; the higher the content of these compounds in patchouli oil, the better the quality [42]. The test results showed no difference in the alcohol content of the patchouli oil produced by the treatment of the condenser cooling water discharge and the drying time of the patchouli leaves. The alcohol content of patchouli oil is 16.84–17.30% (Table 4). Patchouli oil produced in Indonesia has an alcohol patchouli level of <30% because the handling of postharvest material before refining is not proper; the distillation process is not optimal due to the short distillation time and the influence of the origin of the raw material. Therefore, alcohol patchouli content still needs to be increased to expand its market reach [43].

### 3.3. Patchouli Oil Component

The three main components of patchouli oil in all condenser cooling water treatments were alpha-guaiene, delta-guaiene, and patchouli alcohol (Table 5). In general, patchouli essential oil contains patchouli and sesquiterpenoid guaiene as the most exclusive class of compounds [44–48]. The condenser cooling water treatment did not significantly affect the patchouli oil content (Table 5). Differences in the components/composition of patchouli oil qualitatively and quantitatively are caused by differences in environmental factors, areas of origin of samples, differences in harvesting methods, postharvest processing, conditions of distillation, and oil storage [43]. The anti-inflammatory potential of patchouli oil depends primarily on the class of compounds present in its volatile composition. Several guaiane-type compounds exhibit inhibitory effects on proinflammatory cytokines [49]. The guaiene content as an important component in essential oils ranges from 16.14 to 18.90% (Table 5). In addition, patchouli alcohol is also an essential compound in patchouli oil. Patchouli alcohol has been a predominant constituent [48] with the anti-inflammatory, ulcerogenic, bacterial, and fungal activities [50, 51].

## 4. Conclusions

This study confirmed that patchouli oil extraction with the treatment of a condenser cooling water discharge of 1.74 L/min and drying time for 5 days produced the highest patchouli oil yield of 1.4%, the condensate flow rate of 34.67 ml/min, the condenser water temperature of 30.56°C, and the patchouli alcohol content of 16.84%. The greater the condenser water discharge rate, the better the yield and accumulation of patchouli oil recovery obtained. In addition, based on the results of the analysis of the composition of patchouli oil compounds with GCMS, it can be seen that 13 compounds can be detected in patchouli oil. The three main components of patchouli oil in all condenser cooling water treatments were alpha-guaiene, delta-guaiene, and patchouli alcohol.

## Figures and Tables

**Table 1:** The condensate flow rate on several treatments of condenser water discharge and patchouli leaf drying.

Patchouli leaf drying	Condenser water discharge			Average
	1.74 L/min (*A*_1_)	1.08 L/min (*A*_2_)	0.48 L/min (*A*_3_)	
	Condensate flow rate (ml/min)			
5 days (*B*_1_)	34.67^A(a)^	29.33^B(a)^	24.33^C(a)^	29.44^a^
6 days (*B*_2_)	32.33^A(b)^	26.00^B(b)^	20.66^C(b)^	26.33^b^
7 days (*B*_3_)	29.47^A(c)^	24.00^B(c)^	16.00^C(c)^	23.16^c^
Average	32.11^A^	26.40^B^	20.30^C^	

Notes: the numbers followed by the same letter in the same column or the same capital letter in the same line indicate no significant difference by Duncan's multiple range test (*P* < 0.05).

**Table 2:** The condenser water temperature on several treatments of condenser water discharge and patchouli leaf drying.

Patchouli leaf drying	Condenser water discharge			Average
	1.74 L/min (*A*_1_)	1.08 L/min (*A*_2_)	0.48 L/min (*A*_3_)	
	Condenser water temperature (°C)			
5 days (*B*_1_)	30.56^B(a)^	32.47^B(a)^	48.93^A(a)^	37.32^a^
6 days (*B*_2_)	30.90^B(a)^	32.30^B(a)^	49.40^A(a)^	37.53^a^
7 days (*B*_3_)	31.02^B(a)^	32.99^B(a)^	49.50^A(a)^	37.84^a^
Average	30.83^B^	32.59^B^	48.28^A^	

Notes: the numbers followed by the same letter in the same column or the same capital letter in the same line indicate no significant difference by Duncan's multiple range test (*P* < 0.05).

**Table 3:** Oil yield on several treatments of condenser water discharge and patchouli leaf drying.

Patchouli leaf drying	Condenser water discharge			Average
	1.74 L/min (*A*_1_)	1.08 L/min (*A*_2_)	0.48 L/min (*A*_3_)	
	Oil yield (%)			
5 days (*B*_1_)	1.40^A(a)^	1.03^B(a)^	0.34^C(a)^	0.92^a^
6 days (*B*_2_)	1.30^A(b)^	0.90^B(b)^	0.23^C(b)^	0.82^b^
7 days (*B*_3_)	1.23^A(c)^	0.74^B(c)^	0.17^C(c)^	0.71^c^
Average	1.33^A^	0.89^B^	0.24^C^	

Notes: the numbers followed by the same letter in the same column or the same capital letter in the same line indicate no significant difference by Duncan's multiple range test (*P* < 0.05).

**Table 4:** Patchouli alcohol content on several treatments of condenser water discharge and patchouli leaf drying.

Patchouli leaf drying	Condenser water discharge			Average
	1.74 L/min (*A*_1_)	1.08 L/min (*A*_2_)	0.48 L/min (*A*_3_)	
	Patchouli alcohol content (%)			
5 days (*B*_1_)	16.84^A(a)^	17.10^A(a)^	17.14^A(a)^	17.03^a^
6 days (*B*_2_)	16.96^A(a)^	17.11^A(a)^	17.19^A(a)^	17.09^a^
7 days (*B*_3_)	17.30^A(a)^	17.17^A(a)^	17.22^A(a)^	17.17^a^
Average	17.03^A^	17.13^A^	17.18^A^	

Notes: the numbers followed by the same letter in the same column or the same capital letter in the same line indicate no significant difference by Duncan's multiple range test (*P* < 0.05).

**Table 5:** Patchouli oil component on several treatments of condenser water discharge.

Parameter	Formula	Average (%)		
		1.74 L/min	1.08 L/min	0.48 L/min
*beta*-Patchoulene	C_15_H_24_	5.45	5.25	5.03
Seychellene	C_15_H_24_	2.58	2.28	2.72
*trans*-Caryophyllene	C_15_H_24_O	6.76	6.42	6.50
*alpha*-Guaiene	C_15_H_24_	17.15	18.90	16.14
*alpha*-Panasinsen	C_15_H_24_	12.62	13.42	13.16
Naphthalene	C_15_H_24_	12.62	13.42	13.16
*alpha*-Patchoulene	C_15_H_24_	9.06	9.86	9.06
Patchoulene	C_15_H_24_	4.38	3.92	4.25
Azulene	C_15_H_24_	5.65	5.26	5.32
*delta*-Guaiene	C_15_H_24_	16.50	17.58	16.45
Viridiflorol	C_15_H_26_O	3.00	—	1.48
Cycloheptane, 4-methylene-1-methyl-2(2-methyl-1-propen-1-yl)-1-vinyl	C_15_H_24_	—	—	2.66
Patchouli alcohol	C_15_H_26_O	16.84	17.10	17.19

## Data Availability

The data can be found in the National Research and Innovation Agency, Indonesia (https://www.brin.go.id).
